# The HEPS synchrotron unleashes new medical frontiers

**DOI:** 10.1107/S1600577524003874

**Published:** 2024-06-26

**Authors:** Jishizhan Chen, Chong Chen

**Affiliations:** a UCL Mechanical Engineering University College London Torrington Place LondonWC1E 7JE United Kingdom; b Eye Center, Shanghai General Hospital, School of Medicine Shanghai Jiao Tong University Shanghai200080 People’s Republic of China; Bhabha Atomic Research Centre, India

**Keywords:** synchrotron, biomedicine, imaging

## Abstract

The High Energy Photon Source (HEPS) in Beijing achieved a beam energy of 6 GeV. This milestone enables groundbreaking advances in health sciences and various research fields, promising new insights into biological and quantum processes.

In November 2023, the new High Energy Photon Source (HEPS) synchrotron facility under construction in Beijing successfully achieved a designed beam energy of 6 GeV during testing (IHEP, 2023 ▸). This is marked as a major milestone of the HEPS, making it the synchrotron with the highest energy in mainland China and one of the 6 GeV-level synchrotrons worldwide [the others are ESRF-EBS in France (Raimondi, 2016 ▸), APS-U in USA (Hettel, 2021 ▸) and SPring-8 in Japan (Tanaka, 2014 ▸)]. Specifically, among the boosters of the same energy level worldwide, the HEPS has the smallest emittance (meaning the most concentrated beam), and it is the first booster capable of operating in storage-ring mode (IHEP, 2024 ▸*a*). It is expected to become one of the very bright fourth-generation synchrotron radiation sources in the world. At completion by 2025, HEPS will have 14 public beamlines plus one optics test beamline with different focuses (IHEP, 2024*b* ▸) (Fig. 1 ▸). The HEPS therefore represents an opportunity for breakthroughs in various fields, including health science, in which ten emerging topics may benefit: (1) single-particle cryo-electron microscopy (cryo-EM), virus structures and the design of viral inhibitors; (2) dynamic imaging of neural activity, breakthroughs in understanding brain function and disorders; (3) high-throughput X-ray crystallography for drug discovery, rapid identification of potential drug target proteins; (4) advanced phase-contrast imaging for soft tissue, revealing detailed structures of soft tissues without adding contrast agents; (5) time-resolved macromolecular dynamics, including enzyme mechanisms, protein folding and molecular motors; (6) synchrotron-radiation-based immunotherapy research, revealing interactions between nanoparticles and immune cells; (7) molecular imaging for personalized medicine, including personalized analysis of disease processes at the molecular level; (8) nano-bio interface studies, revealing interactions at the nano-bio interface; (9) ultrahigh-resolution 3D imaging of cellular components, revealing new insights into cellular functions and disease mechanisms; and (10) quantum biology, quantum coherence in photosynthesis, enzyme reactions and sensory processes.

## Figures and Tables

**Figure 1:**
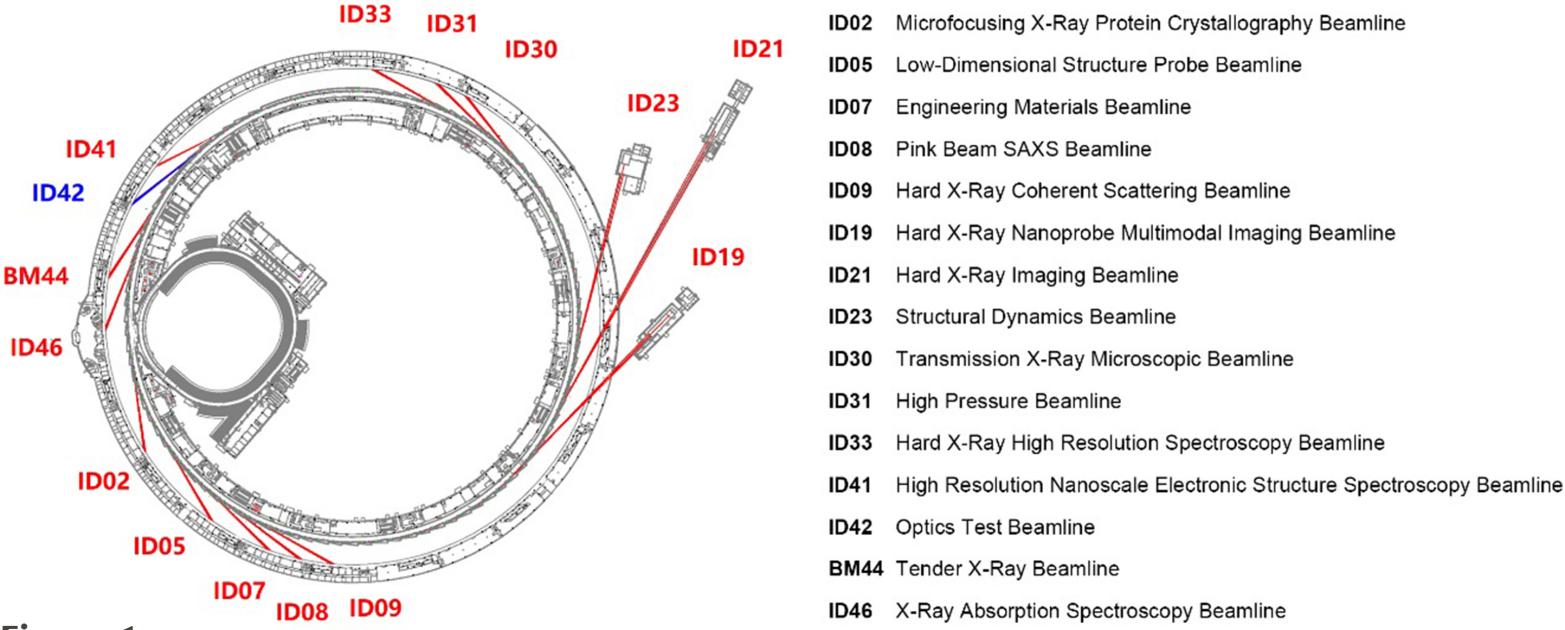
The construction content of the first batch of beamline stations. Adapted from IHEP (2024*b* ▸).
